# Retraction: Identification of XMRV Infection-Associated microRNAs in Four Cell Types in Culture

**DOI:** 10.1371/annotation/f9aef9a9-5488-4492-8b8c-1cdded2e3d79

**Published:** 2013-06-17

**Authors:** Ketha V. K. Mohan, Krishnakumar Devadas, Shilpakala Sainath Rao, Indira Hewlett, Chintamani Atreya

The authors wish to retract this article for the following reasons: 

On re-examination of the data, some errors were identified which contradicted some of the main conclusions of the work. In particular, an error in the calculation of ∆(-) values for miR-193a-3p and miRPlus E1245 was identified, which affects the results reported in Figure 6, and hence the data does not support the conclusions originally drawn regarding the E1245. 

In addition, the interpretation of the presented PCA results is inaccurate since there is no convincing evidence of large or consistent variation related to treatment effect.

Since the experiments were performed only twice, there is no statistical support for the reported miRNA expression level changes during XMRV infection of human cell, and therefore the conclusion that XMRV infection modulates miRNAs in host cells cannot be reported with confidence unless supported by additional experimental validation.

